# David Taylor-Robinson: Medical Microbiologist and Research Pioneer of Sexually Transmitted Infections

**DOI:** 10.7759/cureus.66319

**Published:** 2024-08-06

**Authors:** Simon D Taylor-Robinson, Andrew W Taylor-Robinson

**Affiliations:** 1 Department of Medicine, Busitema University, Mbale, UGA; 2 Department of Public Health, Busitema University, Mbale, UGA; 3 Department of Surgery and Cancer, Imperial College London, St. Mary’s Campus, London, GBR; 4 College of Health Sciences, VinUniversity, Hanoi, VNM; 5 Center for Global Health, University of Pennsylvania Perelman School of Medicine, Philadelphia, USA; 6 College of Health and Human Sciences, Charles Darwin University, Casuarina, AUS

**Keywords:** passionate teacher and mentor, mycoplasmology, non-gonococcal urethritis, ureaplasma urealyticum, gonorrhea and chlamydia, mycoplasma genitalium, genitourinary infection, sexually transmitted infection (sti), medical microbiology, historical vignette

## Abstract

David Taylor-Robinson has been an inspiration to many investigators in the field of sexually transmitted infections (STIs) as, arguably, the father of modern mycoplasmology. Born in 1931, his career as a physician-scientist was initially in virology, researching chickenpox and the common cold, for both of which he made key discoveries at a time when little was known about these conditions. Soon, however, David’s attention turned to bacteriology, developing a passionate interest in mycoplasmas and chlamydia. This gave rise to research collaborations all around the world in marginalized and regional communities, stretching from Tristan da Cunha and Antarctica to the South Pacific and sub-Saharan Africa. He was the discoverer of *Mycoplasma genitalium*, which today is a commonly diagnosed and increasingly antibiotic-resistant pathogen of the genitourinary tract and a significant cause of female infertility. His problem-solving mindset led to research on associations between mycoplasmas with rheumatological conditions and chlamydia with coronary artery plaque formation late into his working life. Throughout his distinguished career, David Taylor-Robinson, affectionately truncated to “DTR” to all who knew him professionally, has been a beloved mentor to hundreds of aspiring scientists, some of whom are now leaders in their field. His open-door policy meant that there was rarely a time when there was no visiting researcher from each of the six inhabited continents under his expert tutelage. A strong work ethic and drive for scientific excellence, allied to his unstinting kindness and jovial demeanor, has provided a source of inspiration to a wide diaspora of research colleagues over more than six decades. This is as much David’s legacy to medical science as the undoubted public health impact of his own pioneering research on STIs.

## Introduction and background

David Taylor-Robinson was born in Bolton in the northwest of England in March 1931. His father, also David, was a South African medical doctor imbued with a punctilious nature and a keen sense of duty. The tense pre-Second World War years saw the family move to Liverpool, where Taylor-Robinson Senior became Professor of Pathology and the head of the city’s public health laboratory service. The Luftwaffe bombing raids on the maritime docks saw a hurried evacuation to North Wales in the autumn of 1940. However, before long, David was back on the ravaged banks of the Mersey, where his experience of post-war hardship helped to instill the resilience and resourcefulness that were to become hallmarks of his medical research career. David’s innate intelligence led him to be easily bored at school and thus to play the classroom joker to amuse himself, much to the exasperation of his disciplinarian father. However, spurred on by healthy rivalry with his academically prodigiously gifted younger brother, Carlton, David “pulled his socks up” and gained entry to the University of Liverpool Medical School in 1949.

## Review

Early medical years

As a committed and diligent student, David enjoyed a reasonably uneventful undergraduate career, punctuated by his love of field hockey, for which he represented Lancashire and later fractured his jaw while playing. He qualified MBChB in 1954, followed by house physician appointments at the Liverpool Royal Infirmary. Proving that the apple does not fall far from the tree, he proceeded to train at Liverpool University in medical microbiology under the watchful gaze of Professor Allan Downie, whose place in medical history is assured for providing the groundwork for the eradication of smallpox worldwide. Perhaps inspired by having such a luminary as a supervisor, David’s doctoral research was itself remarkable. In 1958, this culminated in an MD thesis entitled “Varicella and Herpes Zoster: A Virological Study," a modestly titled seminal work in which he discovered that chickenpox and shingles are caused by the same virus [1].

Foundations of a stellar career

Two years of National Service interrupted David’s virological post-doctoral training in 1959. Rather than take up arms, after boot camp, he was seconded to the UK Ministry of Defence Microbiological Research Establishment at Porton Down in southern England. In these most secretive and high-security Cold War facilities, his microbiology experience was broadened by projects on some of the world's most virulent human pathogens. Fresh out of the military in 1960, David was recruited by the nearby UK Medical Research Council (MRC) Common Cold Research Unit (CCRU) located on the grounds of the Harvard Hospital in Salisbury. Working under the eminent virologists Sir Christopher Andrewes and Professor David Tyrrell, who had discovered the human influenza A virus and the first coronavirus, respectively, these were perhaps halcyon days. He undertook inoculation experiments on healthy volunteers, defining modes of transmission of the common cold and showing that it was not caused by one virus, but many, including a variety of adenoviruses, coronaviruses, and rhinoviruses [2].

Career decisions and developments

**Figure 1:**
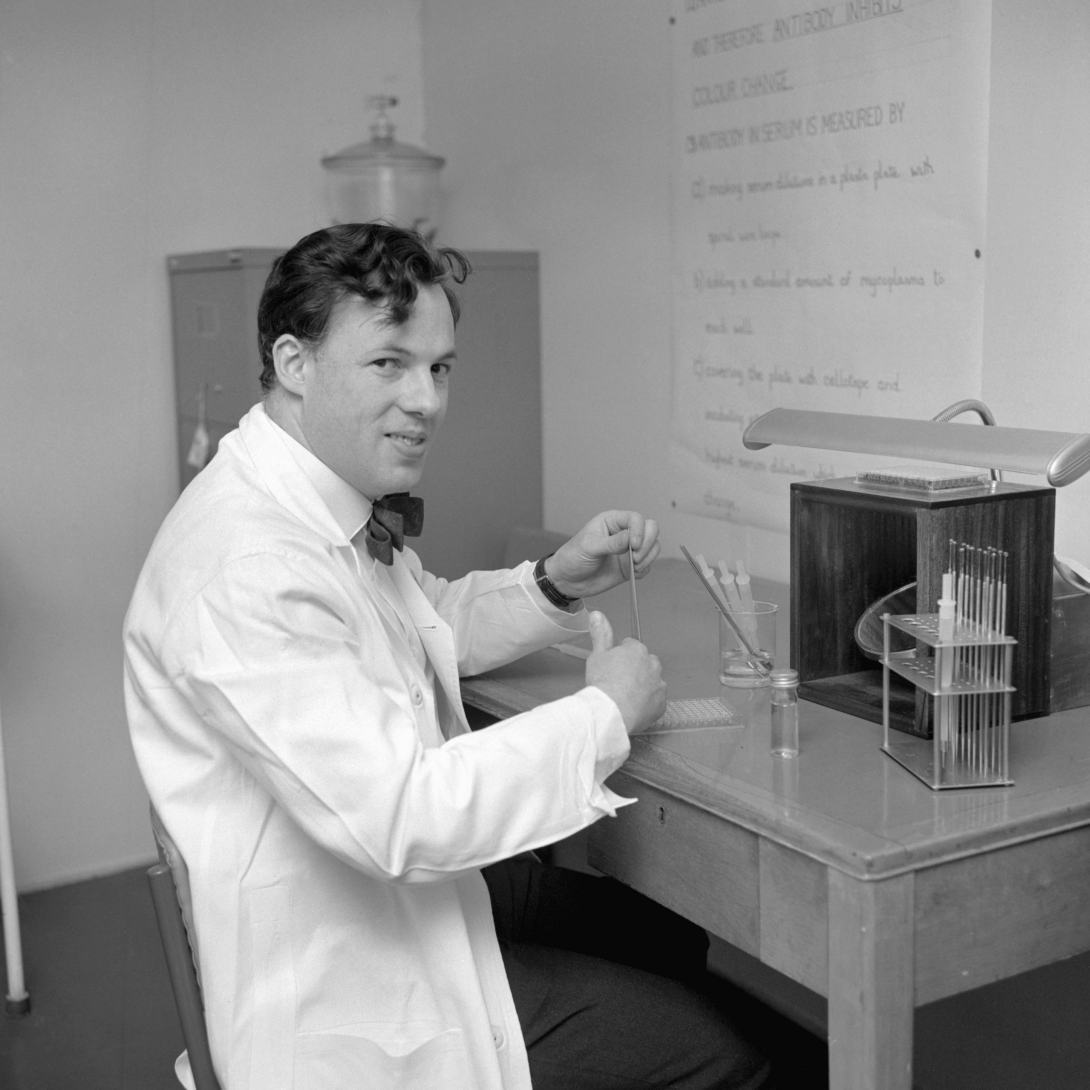
David Taylor-Robinson at the laboratory bench at the MRC Common Cold Research Unit in Salisbury, Wiltshire, UK, in January 1966. PA Images/Alamy, published with permission.

A career-enhancing MRC Fellowship saw David travel to the National Institutes of Health (NIH) in Bethesda, Maryland, in the USA for three years between 1963 and 1965. Working in the Laboratory of Infectious Diseases under the inspiring tutelage of Dr. Robert Chanock, who had recently discovered the human respiratory syncytial virus, he developed a fascination with mycoplasmas, at first respiratory but soon genital, which continued throughout his career [3]. On his return to the UK, he was reemployed by the MRC in Salisbury, becoming a consultant microbiologist in 1966 (Figure 1). However, David’s nascent interest in sexually transmitted infections (STIs), particularly *Mycoplasma hominis* and the recently described Shepherd T-strains (subsequently named *Ureaplasma urealyticum*), saw him at variance with the main ethos of the CCRU [4].

**Figure 2:**
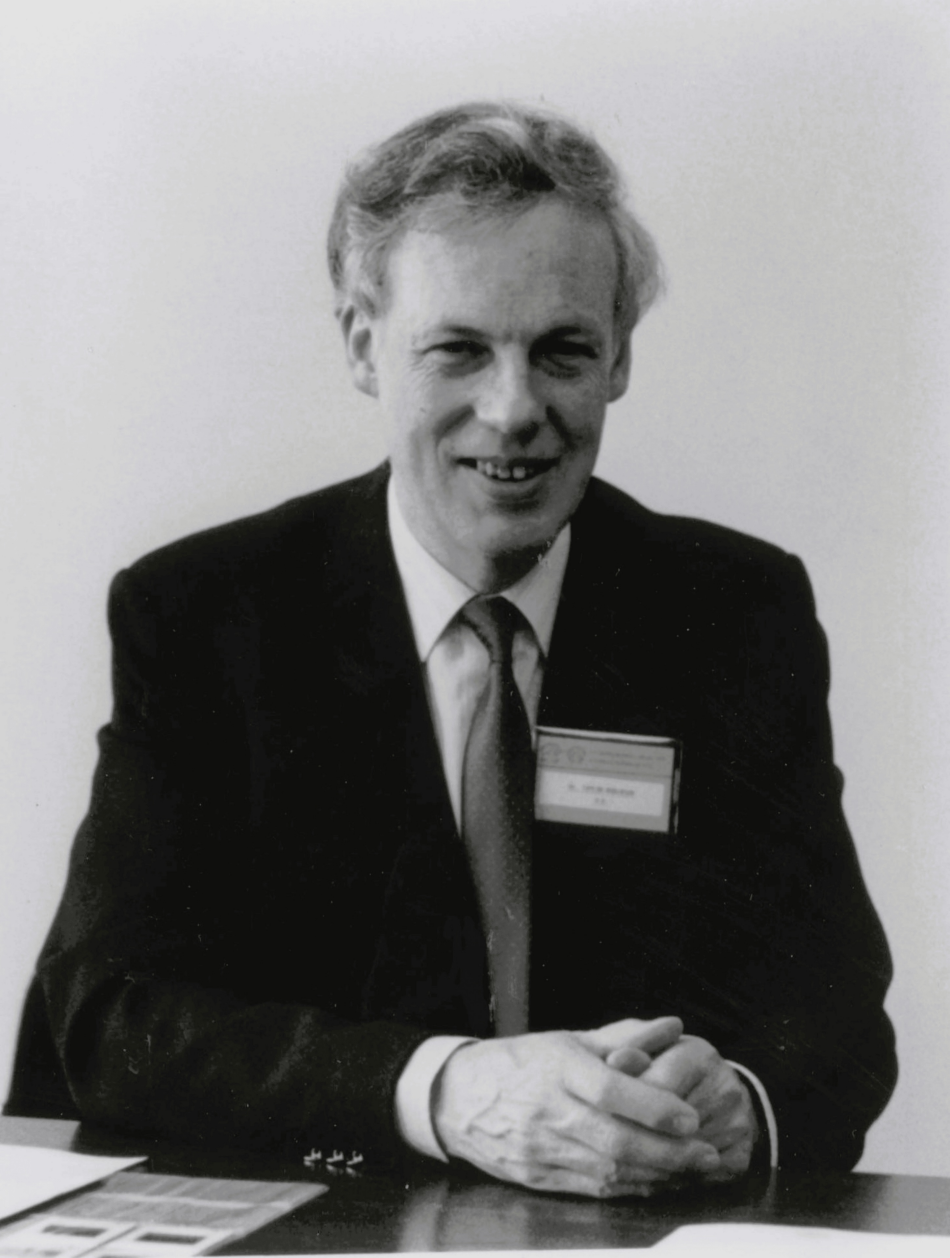
David Taylor-Robinson preparing to speak at the Second International Conference on AIDS held in Paris, France, in June 1986. D. Taylor-Robinson, published with permission.

So it was that, in 1970, David became one of the founding Group Heads at the newly established Clinical Research Centre (CRC) at Northwick Park Hospital in Harrow, in the leafy London outskirts. This brainchild of the MRC brought together clinical practice and academic science in order to better exploit scientific knowledge and developments for the benefit of health care. Now unfettered to explore his scientific intuition in this integrated medical research community, David launched into a series of fundamental laboratory studies, both *in vitro* and using animal models, on mycoplasmas and other species as a possible cause of non-gonococcal urethritis. His inquisitiveness extended from the mid-1970s onwards to seeking an understanding of the pathophysiology of *Chlamydia trachomatis* infection and its transmission and association with pelvic inflammatory disease. Although ethically questionable by today’s standards, 1977 saw the publication of his classic self-inoculation with *U. urealyticum* to demonstrate its pathogenicity; he experienced urethritis with dysuria [5]. David’s subsequent work on STIs in the 1980s and 1990s encompassed clinical trials in marginalized communities, not only men who have sex with men in Europe and North America at the height of the HIV/AIDS epidemic (Figure 2) but also infected young men and women in southern Africa, who at the time were heavily stigmatized [6].

Discovery of *Mycoplasma genitalium*

**Figure 3:**
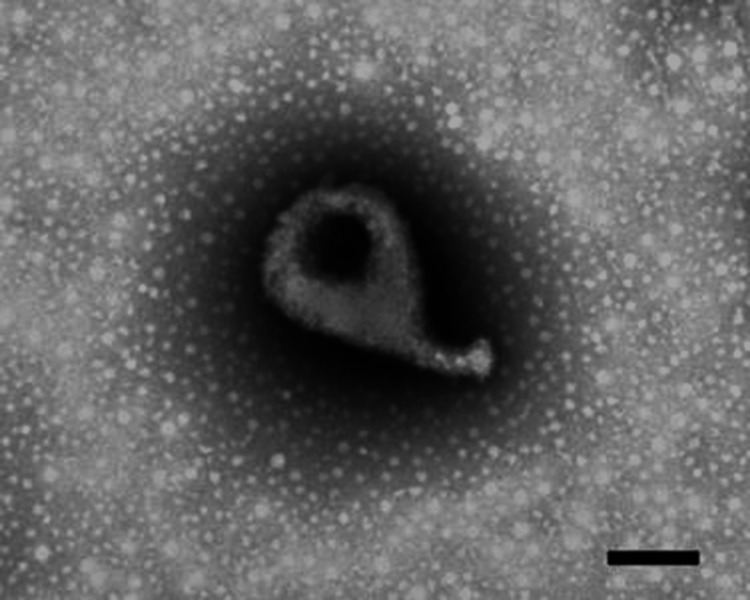
Electron micrograph of Mycoplasma genitalium (bar: 200 nm), showing a classic flask-like appearance. Reproduced from [8]: UK Government Crown copyright 2020, published with permission.

Always nurtured by an inquiring mind, in 1981, David jointly identified (with his good friend Joe Tully) the etiological agent of a hitherto undiscovered STI and cause of acute non-gonococcal urethritis, which they called *Mycoplasma genitalium* [7]. He initially noted the characteristic flask-like appearance on electron microscopy, although he has jokingly likened this to a gourd or butternut squash (Figure 3). *M. genitalium* has subsequently proven to be a globally prevalent STI and, along with chlamydia, an often-neglected cause of female infertility [8]. He took promoting increased awareness of this threat to women’s health very seriously.

International collaborations

Extensive international networking saw, among others, long-term productive coinvestigations with groups at the NIH (principally Dr. Joseph Tully) and Vanderbilt University in Nashville, Tennessee (Professor Zell McGee). David’s research collaborations were fostered by the International Organization for Mycoplasmology (IOM), of which he was elected the founding Secretary-General in 1976, with joint projects stretching from Japan and sub-Saharan Africa to Latin America and the Caribbean. Close collaboration on mycoplasmal studies with Dr. Joseph-Marie Bové from INSERM in Bordeaux, France, led to the award of an honorary doctorate by the University of Bordeaux in 1979. Further recognition came in 1995 from the American Sexually Transmitted Disease Association with what is now the Distinguished Career Award “presented to a person with a long and extraordinary career in the field of sexually transmitted diseases” [9]. In the UK, he gave the eponymous Harrison Lecture in 1994, the highest accolade of the (then) Medical Society for the Study of Venereal Diseases [10].

Imperial College London and later years

Following the politically fueled closure of the CRC and disbandment of its staff in 1990, David was appointed as Professor of Genitourinary Microbiology and Medicine at Imperial College London, consistently rated in the top 10 universities worldwide, relocating with his core group members to the St. Mary’s Campus. There, even in the autumn of his career, David’s lateral thinking impelled him to investigate mycoplasmas as a cause of seronegative arthritis and chlamydia as a cause of coronary artery plaque formation [11].

David formally retired in 1996 after a career almost entirely funded by the MRC. Still going strong at 93 years of age, he remains academically active, taking time and effort to peer review for journals, gaining pleasure in writing insightful editorials, e.g., [12], and caringly mentoring younger generations of microbiologists and genitourinary medicine specialists. His laboratory was always noted for an open-door policy for international collaborators to learn, and it attracted scholars from a plethora of countries from Colombia and Venezuela, through the then Soviet Union, Czechoslovakia, East Germany, and Vietnam, to India, Nigeria, and Japan. David’s love of teaching and desire for knowledge exchange has led to travel to over 140 countries. This habit dies hard, as he was lecturing in Nigeria, Ghana, and Bangladesh shortly before the COVID pandemic of 2020 - a true testament to a knowledge sharer, who in his twilight years continues to educate and enlighten when most mortals would have long since settled down to enjoy the rewards of a well-earned retirement.

A tight-knit team

David has meticulous attention to detail, a quality that lends itself well to scientific research. It should be said, however, that on occasions, this made him intolerant of anything other than what he perceived to be perfection, including himself. Thankfully, for all concerned, those who worked closely with him for a prolonged time were also academic pedants or soon learned to be! Any exasperation soon turned to mirth as he has been blessed by a surreal sense of humor in the vein of another groundbreaker, the comedy writing genius Spike Milligan, so he can definitely see the funny side of things.

David’s work, including the publication of over 750 peer-reviewed papers, would not have been possible without a well-harmonized core team at the CRC, including Drs. Patricia Furr, Eli Fontaine, Alan Johnson, Patricia Munday, Geraldine Taylor, and Brenda Thomas, with each of whom he worked fruitfully for many years. In particular, such was the prolific output and impact of the creative partnership of Furr and Taylor-Robinson, that it was joked that they were to mycoplasmology what the pioneering double act of Laurel and Hardy was to comedy - just without the slapstick. Among others too numerous to mention all, David also worked closely with Professors Myra McClure and Jonathan Weber at Imperial College London, with much-valued clinical collaboration from Drs. Jonathan Ainsworth, Willie Harris, Phillip Hay, Paddy Horner, and Professor Pippa Oakeshott.

A prodigious publication record

In the quarter century between 1970 and 1995, David’s group became established as one of the foremost global centers for research into STIs, with particular expertise in mycoplasmal, chlamydial, and (non-)gonococcal infections. The productivity of his core team, in its various guises, was prodigious, with more than 500 original publications emerging. This is all the more remarkable when one considers that the first draft of most of these were hand-written in David’s incredibly neat but tiny script, always in black ink save for corrections made with his ubiquitous red pen. A brief appreciation here can only scratch the surface of their collective impact on STI treatment, control, and prevention. However, the sheer volume demonstrates admirably that, as with a symphony orchestra, medical research teams need an inspired conductor to channel efforts (and funds) into deliverable results that will benefit humankind.

Family life and the wider world

Although the authors of this article are both David’s sons and each of us has enjoyed a successful academic career in the medical sciences, we did not grow up in an environment focused wholly on his work. In fact, his vivacious wife, Valerie, whom he married in 1959, gently steers his home interests away from medicine, with politics, international relations, history, and travel firmly on the agenda for lively discussion. While the soothing balm of his self-sacrificing spouse has provided a vital antidote to work, the fact that Valerie outranked David during military service, major to captain, is a trump card still played during family debates. He continues to have a great love of sports, both participatory and watching. While field hockey gave way to golf in middle age, his competitive edge means that he is still winning local golf competitions well into his 90s.

A sense of medical history

Of those who could lay claim to be the doyen of modern mycoplasmology, David Taylor-Robinson may be the last man (or woman) standing. In the 1970s till her death in 1985, he befriended Emmy Klieneberger-Nobel, formerly of the Lister Institute of Preventive Medicine in London, who first isolated “pleuropneumonia-like organisms," which we now call mycoplasmas. She described their "fried egg" colony morphology when grown on agar, which due to lacking a cell wall is distinct from other bacteria. It was David who, in his spare time, painstakingly edited her memoir - that of a 1930s Jewish refugee, written in German - for publication in English in 1980. It seems fitting that in 1988, he received the Emmy Klieneberger-Nobel award, the highest honor bestowed by the IOM, “given in recognition of outstanding contributions in research in the field of mycoplasmology." Now, in much the same way that David recognized and helped bring to a wider audience Emmy’s preeminent but easily overlooked pre-war work and from whom the baton was symbolically passed, we acknowledge his lasting legacy to STI research.

## Conclusions

David Taylor-Robinson was a pioneer of medical microbiology research in the second half of the 20th century. As a nod to the history of medicine, in which he took a great interest, David stood on the shoulders of giants of the golden age of bacteriology one hundred years before, making his own profound impact by devoting his long career to studying pathogenic organisms of the genitourinary tract. In so doing, his research considerably furthered our knowledge of the etiology, pathogenesis, and treatment of various STIs, notably non-gonococcal urethritis, bacterial vaginosis, and pelvic inflammatory disease. Moreover, "DTR’s" mentorship and support of the next generations of STI researchers, including many who have progressed to be current leaders in mycoplasmology, ensures that his legacy to medical science will - to use a golfing analogy - last long after he has birdied his final hole.
